# The Effects of Extra Virgin Olive Oil on Alanine Aminotransferase, Aspartate Aminotransferase, and Ultrasonographic Indices of Hepatic Steatosis in Nonalcoholic Fatty Liver Disease Patients Undergoing Low Calorie Diet

**DOI:** 10.1155/2018/1053710

**Published:** 2018-04-17

**Authors:** Farzad Shidfar, Samaneh Sadat Bahrololumi, Saeid Doaei, Assieh Mohammadzadeh, Maryam Gholamalizadeh, Ali Mohammadimanesh

**Affiliations:** ^1^Department of Nutrition, School of Public Health, Iran University of Medical Sciences, Tehran, Iran; ^2^Natural Products and Medicinal Plants Research Center, North Khorasan University of Medical Sciences, Bojnurd, Iran; ^3^Department of Public Health, School of Health, North Khorasan University of Medical Sciences, Bojnurd, Iran; ^4^Department of Nutrition Sciences, Student Research Committee, Ahvaz Jundishapur University of Medical Sciences, Ahvaz, Iran; ^5^Student Research Committee, Cancer Research Center, Shahid Beheshti University of Medical Sciences, Tehran, Iran; ^6^Student Counseling Center, Hamadan University of Medical Sciences, Hamadan, Iran

## Abstract

**Background:**

Coronary artery disease is the most common cause of death in the patients with nonalcoholic fatty liver disease (NAFLD). Studies have shown that there is a strong relation between the increase in the aminotransferase levels and fat accumulation in the liver with cardiovascular complications, independent of all aspects of the metabolic syndrome. This study aimed to examine the effect of virgin olive oil on alanine aminotransferase (ALT) and aspartate aminotransferase (AST) and the severity of steatosis in the NAFLD patients undergoing a weight-loss diet.

**Methods:**

This clinical trial was carried out on 50 patients with nonalcoholic fatty liver (mean age of 45.91 ± 9.61 years, mean BMI of 29.7 ± 0.58 Kg/m^2^) and the subjects were randomly assigned to the olive oil group (receiving the equivalent of 20% of their total daily energy requirement from olive oil) or the control group (with normal consumption of oil) for 12 weeks. All the patients received a hypocaloric diet during the study. At the beginning and the end of the study, the serum levels of ALT and AST and liver steatosis were measured.

**Findings:**

A significant decrease in the level of ALT enzymes was observed in the control group at the end of the study (*P* = 0.004). In the olive oil group, both enzymes decreased compared to baseline measurements (*P* < 0.01). There were significant differences in the ALT and AST levels between the two groups (*P* < 0.02). The severity of liver steatosis did not change significantly during the study.

**Conclusion:**

The consumption of a low calorie diet enriched with olive oil, along with slight weight reduction, reinforces the desired effects of weight loss in improving the levels of the hepatic enzymes.

## 1. Introduction

At present, nonalcoholic fatty liver disease (NAFLD) is the most common cause of elevated serum aminotransferase [1]. In fact, elevated serum alanine aminotransferase (ALT) not only is a consequence of the NAFLD but also predicts the progression of the disease [2]. Recent findings point out that NAFLD may be linked to an increased risk of cardiovascular disease (CVD), which is the most common cause of overall mortality [3, 4]. Many long-term follow-up studies of NAFLD found a strong link between mortality related to the coronary artery disease and NAFLD. Another common reason for mortality in the NAFLD patients is hepatic failure, especially in those with nonalcoholic steatohepatitis (NASH) [5–7]. The increased levels of the liver enzymes including ALT, aspartate aminotransferase (AST), and *γ*-glutamyl transferase (GGT) are the markers of NAFLD and the occurrence of CVD events in both nondiabetic subjects and the patients with type 2 diabetes [8]. Studies have also shown that ALT predicts cardiovascular events, early carotid atherosclerosis [9, 10]. This suggests that NAFLD is associated with coronary heart disease (CHD), independent of the other features of the metabolic syndrome. The studies showed that the prevalence of NAFLD in Iran was relatively high and the people with NAFLD had a higher risk of 10-year CVD events than the individuals without NAFLD [4, 11].

Animal models and human studies suggest that the dietary factors play a key role in the progression of NAFLD. In particular, the amount and type of dietary fat can affect fatty infiltration and lipid peroxidation in NAFLD [12, 13]. There is little research on the effects of the type of dietary fat in NAFLD [14]. Recently, the Mediterranean diet has received attention as a diet that prevents NAFLD and cardiovascular disease. It is known that olive oil, which is rich in the monounsaturated fatty acids (MUFAs), is responsible for the major part of the beneficial effects of the Mediterranean diet [15–17].

Animal studies indicated that olive oil consumption leads to an increase in the release of the triglycerides from the liver and a decrease in the flux of free fatty acids (FFAs) from the peripheral adipose tissue back to the liver [18]. In rats, a diet rich in olive oil led to the remission of hepatic steatosis [19]. In other animal studies, liver damage was found to be decreased in rats receiving olive oil, compared to those given polyunsaturated oil [20]. However, the role of MUFA or olive oil in human NAFLD is yet to be demonstrated.

Some human studies have been conducted on the effects of a high-fat diet (40%) on the serum ALT enzyme and the severity of steatosis in patients with type 2 diabetes [21, 22].

There is only one study on the effects of olive oil in patients with NAFLD who were given a low-fat diet (20% fats) [23]. In this study no significant changes were found in the ALT and AST levels.

Considering the high prevalence of NAFLD in Iran, we have tried to examine the effects of extra virgin olive oil in a normal fat diet (30%) on the serum levels of the ALT and AST enzymes and on the severity of steatosis in the NAFLD patients on a weight-loss diet.

## 2. Materials and Methods

### 2.1. The Subjects

This clinical trial was carried out on 50 patients (19 women and 31 men) with nonalcoholic fatty liver in Tehran, Iran. The mean age was 45.91 ± 9.61 years, and the mean BMI was 29.7 ± 0.58 Kg/m^2^. The clinical inclusion criteria were the increase of the AST and ALT enzymes (U/L < 30 in men and <20 in women), the elimination of all other causes for the increase in the liver enzymes (other liver diseases), age of 20–65 years, BMI of 25–40 kg/m^2^, no use of hepatotoxic medicines, no history of ≥30 gr/d alcohol consumption, no CVD, no diabetes, no pregnancy or breast feeding, no smoking, no consumption of mineral and multivitamins supplements, no consumption of olive products, and lipid-lowering medicines in the last three months. All the subjects gave their informed consent in writing. The trial was approved by the TUMS Research Ethical Committee and was registered in the Iranian website for clinical trials (http://www.irct.ir, code: IRCT201111022709N20).

### 2.2. Sample Size

The sample size was calculated based on the only published paper in this area at the time of this study, which showed that a modified Mediterranean diet (rich in olive oil) caused a reduction in the ALT levels in obese patients with type 2 diabetes [21]. To determine the outcome with type one (*α*) and type two errors (*β*) of 0.05 and 0.20 (power = 80%) and 10% dropouts, 25 subjects in each group were recruited.

### 2.3. The Study Design

The study was a randomized, single-blind trial. The patients were randomly chosen by the qualified experts. The sample was recruited from the visiting patients in the gastroenterology ward of Imam Khomeini's Training and Treatment Hospital in Tehran, Iran. Then, the necessary briefing and the aims of the study were given to the subjects, and they were requested not to use olive oil for 10 days before starting the study. Then, the patients were divided into two groups by using the method of random allocation. The olive oil group received the hypocaloric diet enriched with olive oil (20% of total energy intake) while the control group received the hypocaloric diet with normal fat. None of the participants know about the other group and alternative treatment.

#### 2.3.1. Experimental Design for the Weight-Loss Diet

At the beginning of the study, the weight-loss diet was set with an objective of 5% weight reduction during three months of the study. The daily energy intake recommendations were 50% carbohydrates, 20% protein, and 30% fats for both groups. First, the energy required by each individual was calculated on the basis of their age, weight, and height and the gram quantity of each macronutrient was estimated based on the information. Then, the personalized diet was set, and different food groups and the food-exchanging table were explained to the subjects.

In this diet, 20% of the total fat (30%) was allocated to olive oil or the usual culinary fat, for the olive oil group and the control group, respectively. The remaining 10% of the required daily amount of fat was provided from the other nutrition groups such as dairy, meats, and nuts. Necessary training was given to the patients in the olive oil group for the correct way of consumption of the olive oil at the beginning of the study. The patients in both groups were asked not to change the advised diet and their level of physical activities.

#### 2.3.2. The Preparation Method for Olive Oil

The virgin olive oil used in this study belonged to the Eteka brand (Roudbar, Iran), affiliated to the Khoramshahr Extraction oil company. The required oil dosage was allocated and supplied to each patient every week. In order to reduce the bias in the consumption amount of oil, identical measuring mugs were given to each patient with the required oil amount printed on it.

### 2.4. Measurements

#### 2.4.1. Demographic Data

The demographic questionnaires were completed by interview. The height was measured by using the stadiometer attached to the scale with an accuracy of 0.5 cm without shoes; the weight was also measured by the Seca scale, with an accuracy of 0.5 kg, in the fasting state, and with minimum clothing without shoes. The waist circumference was measured by using the strip meter, with an accuracy of 0.5 cm, with minimum clothing in the standing position at the beginning and the end of the study. The body mass index (BMI) was calculated using weight in kg divided by meters squared. All the patients' medical and drug history were recorded. The level of physical activities was evaluated by using the physical activity international questionnaire.

#### 2.4.2. Dietary Intake

The food record questionnaires were completed over three days (two normal days and one holiday) at the beginning and at the end of the study to estimate the consumption of energy, carbohydrates, protein, fat, vitamins C and E, beta carotene, zinc, selenium, and fiber. The nutrition information was analyzed with the Nutritionist IV software.

#### 2.4.3. Biochemical Assessment

From each patient, 10 cc venous blood sample was taken from the vein in the left arm, after 12–14-hour fasting. The blood samples were taken with the patients in the sitting position, in the Laboratory of Imam Khomeini's Training and Treatment Center, Tehran, Iran. The blood serum was immediately separated using the centrifuge in the 3000th (temperature of 4°C) cycle. The AST and ALT hepatic enzymes in the samples were measured immediately with the enzymatic colorimetric method.

#### 2.4.4. Ultrasound Imaging of the Liver

The severity of the steatosis was measured with an ultrasound in the afternoon, eight hours after a light breakfast. The liver ultrasound was carried out with a 3.5-MHz curvilinear probe by a radiologist. The patients were classified into three groups based on the fat accumulation in their livers, that is, slight, moderate, and severe degrees.

### 2.5. Statistical Analysis

The Statistical Package for the Social Sciences software (version 20, SPSS Inc., Chicago, IL, USA) was used to analyze the data. The Kolmogorov-Smirnov test was carried out to test the normality of the distribution. All variables were reported as mean ± standard deviation (SD). The Chi-Square's test and the independent *t*-test was used for analysing the variables such as physical activity, the type of medicines consumed, the type of edible oil used, and the severity of steatosis between two groups. Within each group, the comparisons were done by the paired-sample *t*-test and by the McNemar's test variables. A *P* < 0.05 was considered statistically significant.

## 3. Results

### 3.1. Demographic Data

Out of the 50 NAFLD patients who participated in this study, 4 patients were eliminated from the olive oil group and 3 patients were removed from the control group. A total of 43 patients completed the trial. The mean age of the subjects was 46.14 ± 8.44 and 45.68 ± 10.8 in the olive oil group and in the control group, respectively. Moreover, the mean BMI in the olive oil group was 29.64 ± 3.93 and in the control group was 29.9 ± 3.77 kg/m^2^. There were no significant differences in gender, age, duration of disease (Table 1), weight, BMI, and waist circumstance (Table 2) between the two groups at the beginning and the end of the study. Given the energy limitation imposed at the beginning of the study, a significant weight reduction of 3.45 kg (4.33%) was observed in the olive oil group and a weight reduction of 2.89 kg (3.54%) was seen in the control group at the end of the study (*P* < 0/001) (Table 2).

As shown in Table 3, there was a significant decreased intake in total energy, carbohydrates, proteins, fat, PUFA, and saturated fatty acids (SFA) in each group and a significant difference in the poly unsaturated fatty acids (PUFA) and the monounsaturated fatty acids (MUFA) intake between the two groups (*P* < 0/001).

### 3.2. AST and ALT Levels and the Severity of Steatosis

There was no significant difference at the beginning of the study in the serum AST and ALT levels between the two groups. At the end of the study, a significant decrease was seen in the ALT and AST levels in the olive oil group (*P* < 0.01), compared to the control group. Moreover, there was a significant difference in both enzymes between the two groups at the end of the study (*P* < 0.05). Although the intragroup liver fat assessment revealed an improvement in both groups (more in the olive oil), there was no significant statistical difference in steatosis between the two groups at the end of the study (Table 4).

## 4. Discussion

In the present study, there was a significant decrease in weight and the ALT and AST levels observed at the end of the study, in both the olive oil group and the control group. Moreover, decrease in the ALT and AST levels in the olive oil group was significantly higher than the control group.

Weight reduction is the first treatment line in the patients suffering from nonalcoholic fatty liver and can motivate the improvement of steatosis and the aminotransferase levels [24]. One study has observed that 5% of weight decrease is enough to decrease the serum's ALT value and to improve steatosis, while a minimum of 9% weight loss is necessary for a significant improvement of NASH [25]. Another study indicated that the patients with more than 7% of their base weight reduction experienced significant improvement in steatosis, inflammation, and the score of NASH tissue activity compared to the patients whose weight loss is less than 7% [26]. Moreover, a weight-loss diet with a goal of 8% initial weight decrease in obese women for a period of 3 to 6 months showed that the effect of weight loss on the improvement of steatosis depends on the rate of weight loss and the initial content of liver fat [27]. In our research, none of the patients had a high or severe steatosis at the beginning of the study and the weight loss was less than 7% in both groups at the end of the study.

Dietary components, particularly the type and the amount of fats, are crucial for liver fat accumulation and are responsible for 15% of the liver fat content. The dietary fats can exert their role in liver steatosis both directly and indirectly (via its influence on adipose tissues) [28]. The studies carried out in this field are limited to the survey of the effects of the modified Mediterranean diet with high fat content and MUFA and its comparison to a low-fat diet in the patients with insulin resistance.

Our research is the first study that has surveyed the effects of the MUFA (from olive oil source) in a diet with normal fat content (30%) administered to the patients with nonalcoholic fatty liver. In one study a balanced-fat diet rich in olive oil rather than sunflower oil after one month leading to a decrease in steatosis and the hepatic enzymes in rats was observed. In human, one study reported that a high-fat (45%) and high-MUFA diet lead to the decrease of the ALT enzymes [21]. These findings are in concurrence with our study. In another study, the patients with NAFLD in the intervention group received a high-MUFA diet (olive oil or canola) for six months [23]. The total content of fat in their diet was 20% of the daily energy intake. Contrary to our study, the daily consumption of 20 g of olive oil had no effect on the serum aminotransferase. The low-fat diet in this study could be a reason for the significant decrease in steatosis. However, the improvement in liver fat content was not adequate for a significant decrease in the aminotransferases.

In a study in the patients with type 2 diabetes it was observed that the percentage of liver fat content in the MUFA-receiving groups with or without exercise had a significant decrease as compared to the groups receiving carbohydrate (CHO) with or without exercise [22].

The beneficial effects of the MUFAs on the hepatic fat content can be explained by the more rapid oxidization of the MUFAs than the saturated fatty acids in the postprandial phase. The more favorable MUFAs deposit in the adipose tissue rather than in the liver following a diet rich in MUFAs may help avoid fat deposition in the liver [29]. In addition, a high-MUFA diet stimulates the activity of lipoprotein lipase more than a diet rich in saturated fats that leads to increase in clearance of circulating triglyceride-rich lipoproteins [30]. In addition to type, the amount of fat also plays a role in the pathogenesis and, probably, in the treatment of fatty liver as well. In our study, the amount of MUFA was less than 20%.

On the other hand, recent studies on nutritional genomics supported a key role of gene-diet interaction in NAFLD development [31]. For example, it is suggested that the obesity-associated (FTO) gene levels in the liver are involved in oxidative stress and lipid deposition, which characterize NAFLD [32]. The level of FTO gene expression is related to the level of dietary macronutrients [33]. Interestingly, the FTO genotype can affect the success of lifestyle interventions in the prevention and treatment of obesity [34].

Moreover, the observed difference may be related to the method of measuring the fatty contents [35]. The NMR (Nuclear Magnetic Resonance) or the spectroscopic golden standard method is used for measuring the existing fat percentage in the liver. It has high precision and accuracy and is considered as the strength of the mentioned study, as described by the researcher. However, in our study, ultrasonography was used because of its cost effectiveness and prevalence. This method has some limitations, including the fact that the results obtained by it depend on the mastery and expertise skills of the operator and the detecting sensitivity of ultrasonography decreases with a degree of fat infiltration less than 30% [2]. Considering the low grade of steatosis in our patients, in contrast to the Nigma study where the patients had higher liver fat, the use of ultrasonography may prevent the obtaining of accurate information on the changes of liver steatosis. Another limitation of this study was sample size and short follow-up duration. A larger group of patients and longer follow-up period are needed to confirm the results.

## 5. Conclusion

In conclusion, the results of this study suggest that normal fat percentage (30%) in a diet containing olive oil (consumption the equivalent of 20% of total calorie intake from virgin olive oil) along with slight weight loss (approximately 5%) reinforces the desired effects of weight loss in improving the levels of the ALT and AST enzymes.

## Figures and Tables

**Table 1:** Demographic characteristics of the study subjects.

Variables	Groups		*P* value
	Olive oil (*n* = 25)	Control (*n* = 28)^††^	
Sex			
Male	13 (61.9%)	13 (61.9%)	0.993^*∗∗*^
Female	8 (38.1%)	9 (40.9%)	
Age (Year)	46.14 ± 8.44	45.68 ± 10.8	0.87^*∗*^
Disease duration (Year)	7.16 ± 2.4	6.91 ± 2.7	0.72^*∗*^
*Type of oil*			0.83^*∗∗*^
Nonhydrogenated oil^†^	15 (71.4%)	16 (73.76%)	
Hydrogenated oil	2 (9.5%)	3 (13.6%)	
Both	4 (9.1%)	3 (13/6%)	

^*∗*^ *P* value reported based on Independent Sample *t*-test; ^*∗∗*^*P* value reported based on Chi-Square test. Quantitative data represented as mean ± SD or median (min-max). Qualitative data reported as frequency (percentage). ^†^In all of the patients using nonhydrogenated oil was sunflower oil. ^††^Was given usual daily consuming oil.

**Table 2:** Anthropometric measurements at baseline and the end of study.

Variables	Groups		*P* value
	Olive oil (*n* = 25)	control (*n* = 28)	
*W* at baseline (cm)	79.65 ± 11	81.65 ± 13.6	0.58^*∗*^
*W* at end-of-trial (cm)	76.2 ± 10.1	78.7 ± 12.9	0.47^*∗*^
*P* value	<0.001^*∗∗*^	<0.001^*∗∗*^	

BMI at baseline (kg/m^2^)	29.64 ± 3.93	29.9 ± 3.77	
BMI at end-of-trial (kg/m^2^)	28.4 ± 3.91	29.13 ± 3.8	0.81^*∗*^
*P* value	<0.001^*∗∗*^	<0.001^*∗∗*^	0.68^*∗*^

WC at baseline (cm)	103.8 ± 10.81	104.18 ± 10.62	0.92^*∗*^
WC at end-of-trial (cm)	100.61 ± 10.1	102.13 ± 10.2	0.63^*∗*^
*P* value	<0.001^*∗∗*^	<0.001^*∗∗*^	

^*∗*^ *P* value reported based on Independent Sample *t*-test; ^*∗∗*^*P* value reported based on Paired *t*-test. Quantitative data represented as mean ± SD or median (min-max). *W* = weight, BMI = body mass index, and WC = waist circumference.

**Table 3:** Dietary intake of study participants, at baseline and after intervention.

Variables	Groups	Before	After	*P*value^■^
		Mean ± SD	Mean ± SD	
Energy (Kcal/day)	Olive oil	2613.8 ± 662.3	1756.5 ± 538	<0.001
	Control	2449.2 ± 723.2	1695 ± 527.1	0.001
	*P*value^*∗*^	0.44	0.7	

Protein (g/day)	Olive oil	79.5 ± 24.28	63.92 ± 23.8	0.001
	Control	82.68 ± 23.18	61.5 ± 22.13	0.005
	*P*value^*∗*^	0.69	0.74	

Carbohydrates (g/day)	Olive oil	374.16 ± 79.1	252.5 ± 53.7	0.004
	Control	334.1 ± 62.6	246.6 ± 49.3	0.005
	*P*value^*∗*^	0.22	0.832	

FAT (g/day)	Olive oil	92.41 ± 55.38	57.3 ± 23.18	0.001
	Control	86.03 ± 36.64	55.33 ± 20.1	<0.001
	*P*value^*∗*^	0.58	0.59	

SFA (g/day)	Olive oil	23.94 ± 9.5	13.67 ± 8.18	0.003
	Control	28.93 ± 8.45	14.03 ± 6.09	0.001
	*P*value^*∗*^	0.411	0.870	

MUFA (g/day)	Olive oil	24.95 ± 9.28	29.27 ± 10.76	0.47
	Control	23.53 ± 10.81	^†^13.45 ± 6.56	0.19
	*P*value^*∗*^	0.873	<0.001	

PUFA (g/day)	Olive oil	37.88 ± 14.63	12.43 ± 4.36	0.002
	Control	32.52 ± 11.8	^†^26.3 ± 8.4	0.003
	*P*value^*∗*^	0.275	<0.001	

Fiber (g/day)	Olive oil	12.49 ± 5.8	10.94 ± 6.5	0.333
	Control	13.4 ± 5.32	11.69 ± 6.23	0.291
	*P*value^*∗*^	0.595	0.070	

Beta-carotene (*µ*g/d)	Olive oil	245/7 ± 57.28	226.68 ± 30.76	0.234
	Control	239.23 ± 95.81	218.82 ± 72.54	0.171
	*P* value	0.708	0.895	

Vitamin E (mg/day)	Olive oil	5.1 ± 3.5	2.53 ± 2.64	0.174
	Control	4.31 ± 3.1	2.16 ± 1.79	0.122
	*P*value^*∗*^	0.634	0.587	

Vitamin C (mg/day)	Olive oil	61.25 ± 42.95	70.95 ± 32.95	0.621
	Control	63.9 ± 22.72	68.05 ± 32.51	0.404
	*P*value^*∗*^	0.863	0.761	

Selenium (mg/day)	Olive oil	0.09 ± 0.04	0.07 ± 0.04	0.134
	Control	0.13 ± 0.17	0.12 ± 0.12	0.823
	*P*value^*∗*^	0.366	0.277	

Zinc (mg/day)	Olive oil	7.04 ± 4.62	10.94 ± 6.5	0.895
	Control	8/04 ± 4.9	11.69 ± 6.23	0.767
	*P*value^*∗*^	0.711	0.615	

^*∗*^ *P* value reported based on Independent Sample *t*-test; ^■^*P* value reported based on Paired *t*-test; SFAs = saturated fatty acids, PUFAs = polyunsaturated fatty acids, and MUFAs = monounsaturated fatty acids.

**Table 4:** Aminotransferase and severity of steatosis at the start and the end of study.

Variables	Groups	Before	After	
		Mean ± SD	Mean ± SD	
				*P* value^■^

ALT (IU/dl)	Olive oil	48 ± 12.9	35.71 ± 11.33	<0.001
	Control	50.82 ± 10.37	46.18 ± 10.26	0.004
	*P* value^*∗*^	0.43	0.003	

AST (IU/dl)	Olive oil	34.53 ± 5.3	26.1 ± 5.4	<0.001
	Control	34.68 ± 8.9	32.28 ± 2.2	0.25
	*P* value^*∗*^	0.94	0.002	

				*P* value^*©*^

Steatosis *N*(%)				
Slight	Olive oil	10 (47.61%)	15 (71.42%)	0.008
Moderate		11 (52.38%)	6 (28.57%)	
Severe		0	0	
Slight	Control	7 (31.81%)	10 (45.46%)	0.17
Moderate		15 (68.18%)	12 (54.54%)	
Severe		0	0	
	*P* value^*∗∗*^	0.23	0.13	

^*∗*^ *P* value reported based on Paired Sample *t*-test. ^■^*P* value reported based on Independent Sample *t*-test. ^*©*^*P* value reported based on McNemar. ^*∗∗*^*P* value reported based on Chi-Square. *P* < 0.05: significant.
